# Herbal medicine: women's views, knowledge and interaction with doctors: a qualitative study

**DOI:** 10.1186/1472-6882-6-40

**Published:** 2006-12-07

**Authors:** Kathryn A Vickers, Kate B Jolly, Sheila M Greenfield

**Affiliations:** 1 Foundation Year One Doctor, Stepping Hill Hospital, Poplar Grove, Stockport, Cheshire SK2 7JE, UK; 2 Department of Public Health and Epidemiology, The University of Birmingham, Edgbaston, Birmingham B15 2TT, UK; 3 Department of Primary Care and General Practice, The University of Birmingham, Edgbaston, Birmingham B15 2TT, UK

## Abstract

**Background:**

There is growing concern that serious interactions are occurring between prescribed/over the counter and herbal medicines and that there is a lack of disclosure of herbal use by patients to doctors. This study explores women's perspectives about the safety of herbal remedies, herb-drug interactions and communication with doctors about herbal medicines.

**Methods:**

Qualitative, cross-sectional study, with purposive sampling which took place in Cheshire, UK. Eighteen in depth semi-structured interviews were conducted with female herbal medicine users aged 18 years and above.

**Results:**

The large majority did not inform their GPs of their use of herbal medicines. This was due to lack of physician enquiry, perception of importance and fear of a negative response. Several women were not aware that herbal remedies could interact with prescribed or over the counter medicines. Of the women who had experienced adverse effects none had reported them, believing them of low importance.

**Conclusion:**

The women had little knowledge about herb-drug interactions and rarely disclosed use of herbal medicines to their doctor. Doctors' communication and openness regarding herbal medicines needs to improve and there should be increased access to accurate information on herbal medicines in the public and health care domain.

## Background

Herbal medicine use is thriving worldwide [1]. In 1998 approximately 20% of participants in a UK survey reported using herbal or homeopathic medicine during the previous 12 months and the sale of herbal medicines grew by 50% in the UK during the period 1995–2000 [2]. Current regulation does not safeguard consumers against unsafe herbal medicine products or require adequate information to be provided by manufacturers [3]. Only some herbal preparations fall under strict regulations in the UK, unlike the stringent examinations required of prescription and over-the-counter drugs (OTC). Herbal medicines are usually marketed as supplements that can be bought OTC either ready made or can also be made up specially for an individual following a consultation with a herbal practitioner and are not subject to such regulation [[1] and [4]], and may "contain potent bioactive substances" [5]. There is growing concern that serious interactions are occurring between prescribed/OTC drugs and prescribed/OTC herbal medicines. A systematic review conducted in 2001 of published herb-drug interactions found evidence of interactions involving widely used herbs with prescribed drugs such as warfarin and garlic, St John's Wort and oral contraceptives [6].

A UK study published in 2004 found one in five patients on warfarin were also taking herbal medicines and 92% were not disclosing herbal medicine use to their doctors. Only 28% were aware that herbal medicines might interact with prescriptions, suggesting that there are public misconceptions regarding the safety of herbal medicine and the need to inform doctors about their use [7]. A UK study investigated whether people would tell their doctor about an adverse reaction to herbal medicine [8] but the reasons for this failure have not been studied. In-depth qualitative studies looking specifically at herbal medicine use in the UK have involved minority groups: Ghanaian women and Sikh immigrants in London. The Ghanaian women were found to have reservations about herbal medicine and its safety and preferred Western medicine when it was available in contrast to the wider usage found among the Sikh population studied [[9] and [10]].

People may use complementary and alternative medicines (CAM) because prescribed medication is not working or has side effects [11]. Suggested reasons for not reporting this use to doctors are that doctors themselves do not address this topic, patients may not think it necessary or be fearful of the doctor's reaction [[12] and [13]]. However the reasons for not reporting herbal medicine use specifically have not been investigated.

Previous studies have indicated primary predictors of herbal medicine use as female gender, white, ethnic origin, high educational status and high income [1,14-16]. Studies have found different age ranges for the highest prevalence of herbal medicine use ranging from young to late middle age [14-16]. Previous UK studies have not investigated in detail the beliefs of the most prevalent herbal medicine users.

This paper reports on a qualitative study of UK female herbal medicine users designed to explore their knowledge, attitudes and behaviour surrounding herbal medicine and the extent to which they disclose herbal medicine use to doctors. This is the first qualitative study to explore the views and knowledge of the most prevalent herbal medicine users in the UK and the first paper to report on reasons why women feel unable to report herbal medicine use and side effects to their doctors. We also report on the reasons why some women think that herbal medicines would not interact with conventional medicines.

## Methods

Purposive sampling [17] was used to obtain a sample of people likely to be frequent users of herbal medicine i.e. white, British females aged 18 years and above [[1] and [14]]. We decided to use qualitative methodology in order to obtain in depth views on herbal medicine and the reasons behind quantitative facts derived from previous studies, for example we know that many people do not report herbal medicine use but we do not know their reasons why. Participants were recruited from two yoga groups (7 women), a therapy centre (4 women) and a women's book club (7 women) in Cheshire. Ethical approval was not required as the data was collected outside of the healthcare setting.

One of the authors (KV) visited groups to describe the research and invite people to be interviewed. A preliminary questionnaire was handed out to identify herbal medicine use (defined for the purposes of the study as any oral medication made from plant leaves, stems, flowers or roots) in the past twelve months. Forms were given out to 70 women and completed forms were collected from the group organisers and participants who wished to be interviewed contacted by telephone, 18 herbal medicine users responded and all wished to be interviewed. All respondents were interviewed in their homes using a previously piloted interview schedule. Interviews lasted from thirty minutes to an hour. The three authors designed the interview by reviewing previous research and looking at areas of herbal medicine taking that had not been tackled and would be suited to qualitative enquiry. The questions were structured in order of topic and were predominantly open-ended with the opportunity for expansion. The themes covered were: reasons for herbal medicine use, sources of advice and information about herbal medicine, interaction with doctors, safety, drug interactions, adverse effects and regulation knowledge. Socio-demographic details (age, ethnicity, occupation) were also collected at the time of the interview.

The interviews were recorded and transcribed. Transcripts were analysed by one of the authors (KV) using analysis 'framework' [18]. A thematic framework of issues and themes was identified according to which the data could be examined and referenced. Indexing was done where the thematic framework was applied systematically and numerically to the transcripts. Charts were drawn up for each key theme taking into account the range of attitudes and experiences. Chart headings reflected key themes from the thematic framework as well as novel themes and condensed themes that had arisen during the coding process. There were entries for several respondents on each chart and a picture of the data as a whole was built up. Finally data was mapped and interpreted by pulling together the key characteristics of the data [18]. For each key theme the range of responses was determined as well as the dominant response. An analytical approach was also incorporated where the number of respondents who had mentioned a theme was counted [17]. This helped to illustrate which issues arose most frequently among this group of women. The way in which certain types of response to one theme on the data charts related to specific responses in another theme was observed. The findings and the conclusions they drew from the data were then discussed amongst the three authors.

## Results

The 18 women interviewed were from a wide age range, all were white British from higher socio-economic groupings (Table 1) and mentioned a range of sources used to obtain information about herbal medicine. The themes and subcategories which emerged from the interviews are grouped under six main headings: (i) motivations for using herbal medicine and the extent of herbal use (ii) reporting herbal medicine use to doctors (iii) co-herbal conventional drug use (iv) beliefs about the testing of herbal products (v) adverse reactions (vi) beliefs about improving information and labelling of herbal medicine. Selected quotes are presented to illustrate the subcategories that arose within these themes.

**Table 1:** Demographic Characteristics of Women Interviewed (*n *= 18)

Demographic	Number
Ethnicity: White British	18

Age (years)	
20–29	5
30–39	1
40–49	3
50–59	7
60–69	2
Mean age	44 years

Socio-economic class*	
1	6
2	7
3	5
*According to national statistics socio-economic classification [19]	

Source of recruitment	
Book club	7
Therapy centre	4
Yoga classes	7

Where herbal information sought*	
Friends	11
Magazines	9
Books	6
None	5
Internet	4
*10 respondents used more than one source of information.	

Where herbal medicine accessed*	
Herbal practitioner	4
Over the counter:	
Health food shop	12
Pharmacy	4
Supermarket	1
Mail order	4
*6 respondents used more than one source	

### Motivations for using herbal medicine and extent of herbal use

Motivations for herbal medicine use were varied but all included at least one of three subcategories: perceived advantages of herbal medicines, beliefs about the disadvantages of conventional health care and medicines (Table 2).

**Table 2:** Summary of Themes and Subcategories Around Motives for Herbal Use

Perceived advantages of herbal medicine (18 women)	Beliefs of disadvantages of doctors and healthcare system (14 women)	Beliefs about disadvantages of conventional medicines (12 women)
1. Natural, holistic (12) 2. No chemicals (12) 3. No/little side effects (7) 4. Personal control (15) 5. Instant access (5)	1. Unable to help some problems (6) 2. Inconvenience of appointments (5) 3. Attitudes especially, emotional issues (4) 4. Negative experiences (4) 5. Time rationing (3) 6. Little faith (3) 7. Embarrassment (3) 8. Not wishing to inconvenience the doctor (3)	1. Chemical (12) 2. Side effects (11) 3. Cause damage (8) 4. Bad past experience (3) 5. Little faith (2)

The majority expressed that part of their motivation for using herbal medicine was due to it being more natural, or holistic or did not involve chemicals.

"I felt better popping this in my mouth than I would a pill. Do you know what I mean? You just feel that it's better for you some how " (interviewee 7).

"I suppose because they are natural plant based and I don't like the idea of putting chemicals into my body " (Interviewee 15).

An important advantage that contributed to preference for using herbal medicines was personal control. This featured in nearly all the interviews and ranged from the belief that it is good to be able to sort things out for yourself, to a strong desire not to be told what to do.

" Because if I take a herbal remedy, I have researched it myself. When I take medicine from the doctor, that is his knowledge, his research, his expertise and I just do as I'm told as a child would" (Interviewee 15).

The second subcategory mentioned by most women was problems with doctors or aspects of the healthcare system. Some women felt doctors were unable to help with certain problems and therefore they looked at alternatives. These problems ranged from minor colds to pleurisy and renal infection. The women contrasted the inconvenience of getting appointments with a doctor with instant access to herbal medicines.

" I have to wait a week before I can see my GP. If I go to a herbalist I can go back in the next day..." (Interviewee 17).

An area of importance for a few women was doctors' attitudes towards them, principally with regard to emotional issues. Past negative experiences with doctors had lead to reluctance to consult and therefore heavy reliance on herbal medicine.

"I mean I did go once. I think I was really quite depressed about 20 years ago and although my doctor did give me antidepressants, his view was that I should get out a bit more and go to tea mornings with the ladies and that would solve my problems. So I didn't like that attitude... I just felt like he thought that I was a silly little woman. ....one time I did go a long time back, it made me very very reluctant to ever go back " (Interviewee 10)

A minority of the women ascribed their reluctance to consult doctors and opt for herbal medicines to time rationing of appointments, little faith in doctors or not wanting to bother doctors.

The third subcategory related to reservations about the side effects and potential damage caused by conventional medicine. Several women believed herbal medicines have no or very little side effects.

"...proper medicine from a doctor. I would class that as proper medicine, different things like that are much stronger and get to the point but there is always side effects, don't they?...Doctor's medicine, where I don't think there are with herbal" (Interviewee 4).

A minority cited bad past experiences and little faith in conventional medicine.

"I was once put on digoxin and I have also had aspirin and all of them had particularly bad effects on me. So I am very very sensitive. So for a very tiny dose of digoxin I was actually having the digoxin side effects..." (Interviewee 13).

The conditions women used herbal remedies for were varied, most commonly were depression, anxiety and stress, colds and flu, premenstrual tension and menopausal symptoms, infections and skin complaints. For women who used herbal remedies for serious conditions this was either due to failed treatment with conventional medicine, or, due to lack of faith in the medical profession. Most bought their herbal remedies over the counter, mainly from health food shops and a few from the pharmacy and mail order. A few visited a herbal practitioner to obtain their herbal medicines (see Table 1). Those who had visited a herbal practitioner tended to hold stronger views about their lack of faith in conventional medical care versus herbal medicine.

"R: What made you decide to start to try herbal remedies".

"I: As I said earlier it was a last resort really and because I kept going back to the doctors and saying something wasn't right and because I was getting very tired and I had lost a lot of weight and I was getting very down about it all, so the doctors couldn't really find anything and so I went (to a herbal practitioner) as a last resort" (Interviewee 11).

"R: So why is it that you decided to go to see a herbalist about this particular problem"?

"I: Em I don't have a lot of faith in GPs or conventional medicine..."(Interviewee 1).

The majority, (14), thought there were definite limits to herbal medicine's capabilities in comparison to conventional medicine. Only a minority, (4), believed that herbal remedies were not limited and could treat serious conditions, including cancer. Most (3) who held this belief had little faith in conventional medicine.

" I think it definitely can be helped by herbs there are a whole host of herbs for cancer..." (Interviewee 13).

### Reporting herbal medicine use to doctors

Most women (15) said they did not usually inform their GPs about herbal medicine use even when prescribed conventional medicines. Six main reasons were given (Table 3), the primary reason being their doctor never asked.

**Table 3:** Reasons Given by Women for Informing or Not Informing Doctors of Herbal Medicine Use

Do not inform doctors of herbal use (15)	Inform doctors of herbal use (3)
Doctor does not ask (12) Does not seem important (5) Belief that taking herbal and prescribed medicine together is harmless especially if for different problems (5) Past negative attitudes when informed (4) Fear of response, being treated differently (3) Doctors not interested or have no knowledge of herbs (2)	Doctor open minded (2) Good relationship with doctor (1) Feel it is important to inform as they do not want to mix drugs (1)

Numbered in order of frequency mentioned by the women.

"R: If you go to the doctors do you tell the doctor you are taking a herbal medicine?

I: No. I don't think the doctor has ever asked " (Interviewee 3).

The second reason was because it never occurred to the women or seemed unimportant. Some believed there was no danger in taking herbal and prescribed medicine, especially if the medications were for different problems. A few described past negative experiences on disclosure of use, fear of the doctor's response and the belief they would not be interested.

I: " ...I did mention positively that I'm actually finding starflower oil helping you know. And I don't always feel that it's received very well".

R: "Right".

I: "I almost feel like I'm a naughty girl doing that " (Interviewee 1).

Two women said they would not even mention herbal medicine use to their doctors if they were aware that they could interact with their prescribed medication due to fear of the response they might receive.

I: "Interact badly, do you mean, or well in any way"?

R: "Yes in any way"?

I: "I don't know. I am very worried about talking to GP's about herbal medicine. My sense in the past has been that they are just not interested or they are not up for it or shouldn't be messing around with it " (Interviewee 1).

For the minority who regularly informed their doctor of herbal medicine use, the primary reason was because they had a good relationship with their doctor who was open-minded.

"Because I think I have quite a good relationship with my doctor.... she is much more open now I think to alternative " (Interviewee 6).

### Co-herbal conventional drug use

Seven women were not aware that some herbal medicines could interact with certain prescription drugs. Eleven said they would use both herbal and conventional medicines over the same time period; most (8) believing that if the medicines were for different conditions it would be safe. Others (2) thought if the conditions were not serious it would not matter.

"No I think ginseng is quite mild so it is unlikely to react with medication. If I was taking herbal then I wouldn't take prescribed for the same problem. It also depends on what is wrong with you. I don't think that things I take for say the immune system or detox or to boost your mood would react with something like antibiotics " (Interviewee 17).

Two respondents were unaware they were co-ingesting herbal medicine and prescription medicines as they had not realised the oral contraceptive pill was a prescription medicine. In addition, every respondent said they used over-the-counter medicines at some time, which could also lead to potential mixing and interactions.

### Adverse reactions

The majority (13) said they had never experienced any adverse reactions to herbal remedies. For those that had (5) the commonest herbal medicine was St John's Wort that had been bought OTC. Other adverse effects were felt to be due to overdose or prolonged use of a potentially damaging OTC herbal medicine. Those who had experienced detrimental effects said they had not informed anyone due to underestimation of their importance.

R: "With the eyes did you actually see anyone about that at all, about the symptoms you were experiencing"?

I: "No... I saw a tiny little paragraph in a magazine that commented on people having trouble with eyes on St John's Wort ... it said not to worry come straight off it " (Interviewee 1).

Those respondents who had never experienced adverse reactions described a range of potential sources where they would get help if it occurred. Most (7) said they would inform their doctor but a few (3) of these would only do so if they were very ill, otherwise they said they would consult a herbal shop. Some (4) said they would consult a herbal shop for all reactions including severe ones. A few said they would take no action as they would be afraid to admit their herbal medicine use to health professionals.

"I don't know because could you go to a pharmacist and go erm, I've taken this? I don't know they might be quite dismissive of you. I don't know " (Interviewee 7).

Only one respondent said that they would also inform the manufacturer.

### Beliefs about herbal testing

On the theme of safety, few (3) believed herbal medicines were completely harmless but the vast majority (14) thought they were relatively safe.

"You tend to think that they are very safe. Which could be quite naïve but just the word herbal you think ah it must be good for you, it must be safe " (Interviewee 7).

Many (10) believed there is testing done on herbal medicines for safety. The remainder (8) did not know whether there were tests. Several (8) respondents believed that herbal medicines are tested to see if they cure disease and some (6) believed they are tested for consistency. The majority (12) understood there is not strict regulation of herbal products. Some (3) believed this is due to herbal medicines being safe.

"Well I think if people are grownups they should be allowed to take what they want...

You don't hear about people dying in droves from herbal medicines " (Interviewee 14).

The majority (6) of those that were aware regulation is not stringent said they would like it to improve. The remainder (2) felt they would not want improvements due to fear of being denied access.

"I think the EU is trying to sweep a lot of them off our shelves which I am not particularly happy about " (Interviewee 14).

### Beliefs about improving information and labelling of herbal medicines

Virtually every interviewee said they would like more information on aspects of herbal remedies including improvements on labelling and comprehensive leaflets included with products, which would state what the herbal medicines are for and any side effects or contraindications.

R: "How do you think the labelling could be improved"?

I: "With more advice, what it is recommended for and also contraindications which you do get on normal medications " (Interviewee 15).

Other areas suggested for improvement were: information about interactions and action to take if a reaction occurred (5); poor labelling of dose and duration (4); absence of ingredient lists on their Chinese herbal medicines (2), which was of great concern for one interviewee who had allergies; the need for simpler language on leaflets and larger print (2); trials on herbal medicines and information on the state of present regulation and testing (7).

## Discussion

From a medical perspective, simultaneous use of herbal and conventional drugs has the potential to be a serious threat to health [1] and lack of communication between users and doctors regarding the issue contribute to this [7]. This study was therefore essential to get the lay perspective on these issues and to formulate an understanding of what is impeding discussion between doctors and herbal medicine users. Previous studies have investigated people's reasons for seeking CAM generally [[11] and [13]] and reasons for not reporting CAM use to doctors [[12] and [13]]. This is the first UK study to explore in depth the beliefs and motives for using herbal medicine, co-herbal conventional drug use and the reasons for not reporting herbal medicine use to doctors, of women whose socio-demographic characteristics reflect those of the main users of herbal medicine documented in previous studies [14-16]. Although the results of the study are not generalisable to the UK population as a whole, due to the small-scale opportunistic sampling approach used, the study has elicited some of the reasons behind non-disclosure and lack of adverse effect reporting.

Motives for herbal medicine use among the women interviewed for the current study were centred essentially on the contrasting advantages of herbal medicine and disadvantages of conventional medicine. The main factors mentioned were unsuccessful conventional treatment and concerns about side effects [11] and lack of satisfaction with orthodox medicine and its failure to take a holistic approach [[20] and [21]] versus the belief that herbal medicine is natural and free from chemicals and they are able to maintain their personal control by taking it instead of being told what they should do by doctors.

The discovery that many of the study participants believed that herbal medicines are not chemical was related to their beliefs that herbal medicines are relatively safe. Similar findings arose in studies into CAM therapies in general where the belief they are 'natural' appears to be synonymous with 'safe' [22]. This suggests that awareness needs to be raised that herbal medicines may contain powerful and potentially toxic ingredients and therefore need to be treated by users in the same way as conventional medicines. There was a lack of awareness of the extent of herbal medicine testing among the study participants and a desire for better labelling of products. This highlights the need for the level of testing to be stated on the product. This has now been introduced in Canadian products to guide consumers [23].

It is evident from this research that consumer awareness of interactions between herbal medicine and conventional medicine is lacking and this has the potential to cause serious interactions. This supports a previous study conducted in 2004 in the UK involving patients taking warfarin and herbal medicines, where only 28% thought that they may interact [7]. It is of paramount importance therefore that awareness is raised among both the public and health care professionals that herbal, prescription and OTC medicines can interact. It needs to be emphasised that even if the medicines are for different conditions or minor illnesses there could still be potential for serious interactions.

Discussion of herbal medicine use with doctors was uncommon among the study participants despite the fact that interviewees were from higher socio-economic groups who are more likely to be knowledgeable about health related matters and more proactive in their interactions with doctors [24]. The reasons for non-disclosure of herbal medicine use shown in the current study, mirror reasons for non-disclosure of CAM in previous studies [[12] and [13]] such as that doctors fail to ask about herbal medicine usage or patient lack of awareness or fear of the doctor's response. It appeared that no doctor had ever asked any of the women interviewed in the current study if they use herbal medicine but nearly all said they would disclose use if they were asked. The perceptions of the women in the current study regarding doctors' attitudes towards herbal medicines were predominantly negative. Therefore doctors' awareness that they need to ask about herbal medicine needs to be raised and that their attitudes and communication skills will influence patients' willingness to discuss herbal medicines. Safety and relationships with their patients are likely to be better maintained if doctors are willing to enter into non-judgmental dialogues with patients about herbal medicines and take on board why herbal medicine is meaningful to them and allow their patients to maintain personal control in decisions about treatment options.

A UK study found that people would be less likely to report adverse reactions to herbal compared to conventional medicine and this suggests that strategies need to be put in place to ensure communication of this type of information [8]. It was discovered by this research that the reasons for not reporting adverse herbal reactions were lack of realisation of importance and fear of informing health professionals. This could cause serious morbidity and delayed treatment. This points to the requirement for patient information at GP practices that includes encouragement to inform their GPs of herbal medicine reactions and explain that they will be treated with respect and understanding. It is only through reporting reactions that effective monitoring can occur and due safety measures be implemented.

## Conclusion

This research has gained insight into areas regarding simultaneous herbal and conventional drug use among UK women and their interaction with doctors about these issues. It has elicited some reasons behind non-disclosure and lack of adverse effect reporting It was discovered by this research the reasons for not reporting adverse herbal reactions were specifically to do with herbal medicine as opposed to CAM in general. The study has however shown similarities to studies that have looked at CAM in general in the UK [[11,20] and [21]].

Discussion of herbal medicine use with doctors appears to be infrequent and the key limiting factors uncovered appear to be the lack of realisation of importance, doctors' failure to ask, communicate and be approachable. Women's lack of knowledge of herb-drug interactions and the need to report adverse effects is of great concern, especially amongst an educated group. This level of knowledge may be reduced still further in a more socio-economically diverse group. It seems communication between health professionals and patients is the key to overcoming many of these issues and willingness to take each other's beliefs on board is the only way to form a safe alliance between conventional and herbal medicine.

## Competing interests

The author(s) declare that they have no competing interests.

## Authors' contributions

KV, KJ, SG contributed to the conception and design of the study.

SG and KJ supervised KV during the study.

KV collected the data, analysed and interpreted the data.

KV wrote the first draft of the research and was involved in the redrafting.

SG and KJ revised the drafts critically.

All of the authors have read and approved the final draft.

## Pre-publication history

The pre-publication history for this paper can be accessed here:


